# Supplementary material for: Modeling the Immune Response for Pathogenic and Nonpathogenic Orthohantavirus Infections in Human Lung Microvasculature Endothelial Cells
Source: Viruses. 2023 Aug 24;15(9):1806. doi: 10.3390/v15091806 (PMC10535571; doi:10.3390/v15091806)
Supplement: Supplementary file 1 [file viruses-15-01806-s001.zip › viruses-2503454-supplementary.pdf]

Table S1. Results of nonlinear least square fits to the solutions of the system of ODEs by gene and virus. The values in parentheses below each parameter are margins of error corresponding to a 95% confidence interval. The SSE is the sum of squared errors, while t.max and y.max give, respectively, the time at which the peak occurs and its value.

Gene	Virus	Parameter Estimates			Curve Statistics		
		a_i1	a_i2	r_i	SSE	t.max	y.max
CCL5	ANDV	1.4×10^{-11}<br>($\pm 7.4 \times 10^{-3}$)	5.0×10^{-3}<br>($\pm 1.1 \times 10^{-2}$)	9.1×10^{-2}<br>($\pm 9.2 \times 10^{-2}$)	342.0	53.8	120.5
	HTNV	9.1 × 10−12<br>(±3.3 × 10−3)	2.2×10^{-3}<br>($\pm 4.5 \times 10^{-3}$)	9.0×10^{-11}<br>($\pm 2.6 \times 10^{-2}$)	219.1	72.0	168.0
	PHV	1.4×10^{-11}<br>($\pm 3.8 \times 10^{-3}$)	1.9×10^{-3}<br>($\pm 5.2 \times 10^{-3}$)	2.2×10^{-2}<br>($\pm 4.9 \times 10^{-2}$)	220.0	64.1	90.7
CXCL10	ANDV	3.1×10^{-9}<br>($\pm 4.6 \times 10^{-2}$)	5.9×10^{-2}<br>($\pm 9.9 \times 10^{-2}$)	3.2×10^{-1}<br>($\pm 3.0 \times 10^{-1}$)	574.8	40.3	603.4
	HTNV	7.4×10^{-3}<br>($\pm 1.4 \times 10^{-2}$)	1.6×10^{-3}<br>($\pm 1.9 \times 10^{-2}$)	3.3×10^{-2}<br>($\pm 4.1 \times 10^{-2}$)	5430.0	47.6	399.8
	PHV	7.8×10^{-3}<br>($\pm 1.3 \times 10^{-2}$)	4.0×10^{-12}<br>($\pm 1.8 \times 10^{-2}$)	4.2×10^{-2}<br>($\pm 5.4 \times 10^{-2}$)	4090.0	45.5	319.3
CXCL11	ANDV	3.4×10^{-12}<br>($\pm 2.4 \times 10^{-2}$)	1.5×10^{-2}<br>($\pm 3.6 \times 10^{-2}$)	9.5×10^{-2}<br>($\pm 1.1 \times 10^{-1}$)	6390.0	45.2	376.4
	HTNV	8.6×10^{-12}<br>($\pm 1.7 \times 10^{-2}$)	8.6×10^{-3}<br>($\pm 2.2 \times 10^{-2}$)	1.9×10^{-2}<br>($\pm 3.6 \times 10^{-2}$)	10100.0	55.2	454.2
	PHV	2.8×10^{-3}<br>($\pm 7.9 \times 10^{-3}$)	5.3×10^{-3}<br>($\pm 1.1 \times 10^{-2}$)	2.5×10^{-2}<br>($\pm 2.1 \times 10^{-2}$)	2070.0	51.9	398.7
IDO1	ANDV	7.7×10^{-13}<br>($\pm 5.5 \times 10^{-5}$)	1.2×10^{-3}<br>($\pm 7.0 \times 10^{-5}$)	1.1×10^{-3}<br>($\pm 6.3 \times 10^{-4}$)	0.1	44.1	22.6
	HTNV	7.8×10^{-9}<br>($\pm 1.2 \times 10^{-3}$)	6.8×10^{-4}<br>($\pm 1.8 \times 10^{-3}$)	8.0×10^{-2}<br>($\pm 9.2 \times 10^{-2}$)	21.1	46.6	19.4
	PHV	2.3×10^{-12}<br>($\pm 5.2 \times 10^{-4}$)	4.0×10^{-4}<br>($\pm 7.4 \times 10^{-4}$)	7.2×10^{-2}<br>($\pm 5.9 \times 10^{-2}$)	4.4	46.6	12.2
IFNB1	ANDV	3.7×10^{-13}<br>($\pm 1.6 \times 10^{-2}$)	6.4×10^{-3}<br>($\pm 2.9 \times 10^{-2}$)	2.0×10^{-1}<br>($\pm 4.6 \times 10^{-1}$)	336.3	41.9	95.7
	HTNV	4.3×10^{-5}<br>($\pm 7.8 \times 10^{-4}$)	6.6×10^{-4}<br>($\pm 1.0 \times 10^{-3}$)	3.0×10^{-2}<br>($\pm 2.5 \times 10^{-2}$)	18.7	51.9	32.1
	PHV	6.6×10^{-4}<br>($\pm 9.0 \times 10^{-4}$)	3.1×10^{-4}<br>($\pm 1.2 \times 10^{-3}$)	2.7×10^{-2}<br>($\pm 2.1 \times 10^{-2}$)	26.1	49.8	46.3

Table S2. Fold change (log_2) of each gene at 12, 24, 36, 48, 60, and 72 hpi following infection by ANDV, HTNV and PHV.

	ANDV						HTNV						PHV					
	12	24	36	48	60	72	12	24	36	48	60	72	12	24	36	48	60	72
ARG1	0.00	0.00	0.00	0.00	0.00	0.00	0.00	0.00	0.00	0.00	0.00	0.00	0.00	0.00	0.00	0.00	0.00	0.00
CASP3	0.41	0.04	0.55	0.59	0.51	1.08	0.14	0.01	0.03	0.34	0.55	1.19	-0.22	0.14	0.22	0.69	0.76	1.19
CASP7	0.20	0.03	0.23	0.25	0.24	0.15	0.23	0.02	0.16	0.25	0.24	0.16	0.18	0.24	0.39	0.46	0.57	0.49
CASP8	0.36	0.50	1.21	1.15	1.13	1.08	0.30	0.39	1.01	1.10	1.17	1.14	0.28	0.66	1.36	1.44	1.52	1.59
CCL2	0.28	0.16	0.33	0.52	0.43	0.31	0.27	0.22	0.29	0.43	0.61	0.33	0.25	0.19	0.64	0.70	0.83	0.81
CCL22	0.79	0.46	0.90	0.34	-0.41	0.68	0.98	0.26	0.72	0.26	-0.21	0.85	0.67	0.98	0.78	1.20	1.24	1.30
CCL5	0.20	-0.16	0.53	0.36	0.30	1.05	-0.23	-0.28	0.07	0.14	0.32	1.30	-0.21	0.50	0.17	0.46	0.49	0.68
CCR7	0.19	0.91	4.30	6.89	6.68	6.23	-0.06	0.76	2.98	5.80	6.65	6.37	0.24	1.09	3.41	6.24	7.18	7.45
CD14	-0.03	-0.06	0.50	0.20	0.30	1.05	-0.38	-0.35	-0.18	0.00	0.36	1.28	-0.25	0.60	0.10	0.53	0.42	0.73
CD200R1	0.05	0.25	0.27	0.25	0.20	1.06	-0.53	-0.21	-0.25	-0.10	0.14	1.27	-0.89	0.11	-0.41	0.44	0.06	0.73
CD274	0.11	-0.30	0.32	0.22	0.24	0.59	-0.52	-0.38	0.18	0.04	0.28	0.80	-1.01	-0.67	-0.44	0.03	0.34	0.72
CD80	-0.04	0.67	2.41	1.92	1.33	1.42	-0.05	0.81	1.81	1.54	1.55	1.59	-0.09	1.06	1.93	1.91	2.18	2.19
CD86	0.25	-0.29	0.54	0.61	0.61	0.91	0.02	-0.15	-0.16	0.34	0.68	0.98	0.03	0.22	0.39	0.78	0.81	0.86
CXCL10	0.34	-0.19	0.59	0.70	0.49	0.66	0.37	-0.11	-0.01	0.47	0.58	0.87	0.22	0.29	0.51	0.72	0.71	0.85
CXCL11	2.97	5.18	9.04	8.88	7.29	5.94	1.04	5.39	8.06	8.25	7.71	7.67	0.63	5.40	8.03	8.77	8.18	8.13
IDO1	1.84	4.32	7.67	8.73	7.66	6.71	0.69	4.52	7.70	8.69	8.44	8.35	0.08	3.90	7.39	8.92	8.57	8.73
IFNA1	0.35	0.95	3.93	4.66	3.32	2.11	0.04	0.43	2.95	3.84	3.07	2.72	0.09	0.95	3.48	4.53	3.53	3.16
IFNB1	0.08	-0.16	0.43	0.56	0.58	0.96	-0.04	-0.11	0.12	0.37	0.55	1.14	-0.09	0.44	0.40	0.76	0.79	0.93
IFNG	0.59	1.57	6.18	6.56	4.61	3.25	0.12	2.69	4.85	5.61	5.35	5.15	-0.27	2.20	4.02	5.09	4.85	4.67
IL10	0.14	-0.21	0.25	0.39	0.63	0.92	-0.05	-0.33	-0.10	0.19	0.33	0.83	-0.21	0.06	0.20	0.52	0.83	0.80
IL12A	0.18	-0.06	0.57	0.40	0.51	1.03	-0.07	-0.04	0.15	0.23	0.45	1.23	-0.13	0.46	0.34	0.56	0.46	0.86
IL15	0.43	-0.01	1.27	1.69	1.19	1.30	0.14	-0.07	0.87	1.30	1.25	1.36	-0.18	0.03	0.85	1.61	1.53	1.67
IL1A	0.32	0.69	1.80	1.81	1.69	1.45	0.13	0.58	1.52	1.59	1.91	1.69	0.17	0.84	1.88	1.89	1.99	1.86
IL1B	0.42	0.20	1.64	1.43	0.78	0.50	0.39	0.21	1.23	0.82	0.60	0.43	0.11	0.50	1.37	1.54	1.59	1.41
IL1RN	0.56	0.18	0.83	1.01	0.70	0.49	0.52	0.28	1.08	0.87	0.57	0.50	0.28	0.55	1.11	1.74	1.85	1.46
IL6	0.16	-0.17	0.56	0.68	0.48	1.18	-0.15	-0.21	0.19	0.47	0.56	1.35	-0.07	0.58	0.32	0.66	0.57	0.85
IL8	0.59	0.38	1.25	0.98	1.00	1.21	0.49	0.40	0.62	0.58	1.00	1.19	0.25	0.48	0.84	0.93	1.24	1.57
IRF3	0.26	0.16	-0.09	0.15	-0.31	-0.67	0.34	0.53	0.49	0.08	-0.60	-0.86	0.05	-0.07	-0.26	-0.55	-0.35	-1.02
IRF7	0.21	0.19	0.14	0.08	0.23	0.37	0.16	0.14	0.15	0.05	0.28	0.44	0.14	0.25	0.46	0.33	0.50	0.61
ITGAM	1.59	3.53	4.51	4.51	4.46	4.41	0.64	3.04	4.23	4.42	4.59	4.55	0.30	3.19	4.70	4.63	4.93	5.05
NFKB1	0.16	-0.25	0.59	0.68	0.53	1.11	-0.05	-0.20	0.10	0.47	0.56	1.28	-0.05	0.59	0.40	0.71	0.99	0.94
NOS2	0.14	-0.06	0.02	-0.02	0.08	0.35	0.16	-0.09	-0.07	-0.02	0.08	0.45	0.12	0.29	0.26	0.32	0.29	0.47
PPARG	0.06	-0.27	0.47	0.29	0.45	1.06	-0.06	-0.59	0.05	-0.07	0.54	1.28	-0.16	0.26	-0.07	0.41	0.60	0.81
TGFB1	-0.09	-0.23	0.18	0.20	0.45	0.60	-0.14	-0.31	0.03	0.43	0.44	0.66	0.09	0.19	0.51	0.60	0.53	0.42
TLR3	0.14	-0.10	0.04	-0.03	0.15	0.46	0.06	-0.05	-0.03	-0.02	0.18	0.49	0.17	0.43	0.32	0.41	0.50	0.59
TLR4	0.12	0.06	0.32	0.37	0.48	0.43	0.11	0.02	0.21	0.41	0.51	0.44	0.04	0.22	0.62	0.56	0.75	0.73
TLR7	0.98	2.60	4.89	5.83	5.11	4.56	0.37	2.46	4.52	5.56	5.57	5.14	0.03	2.18	4.92	5.82	5.77	5.72
TNF	0.09	0.13	0.29	0.21	0.44	0.65	0.10	0.07	0.03	0.12	0.43	0.68	0.00	0.40	0.56	0.48	0.61	0.89
VEGFA	0.12	-0.26	0.47	0.54	0.55	0.97	-0.07	-0.17	0.12	0.51	0.62	1.16	-0.04	0.46	0.32	0.71	0.75	0.93

Table S3. Levels of secreted cytokines, chemokines, and interferon proteins by HLMVECs from four donors at 48 and 60 hpi following infection by ANDV, HTNV and PHV (log_2 pg/ml)

hpi	Protein	Virus	2559(M)	2572(M)	2551(F)	438Z013.1(F)
48	CCL5	ANDV	10.6	11.3	11.3	11.3
		HTNV	10.0	11.3	11.3	10.6
		PHV	10.0	9.6	10.6	8.0
	IFN-β	ANDV	10.6	10.3	12.0	11.0
		HTNV	10.3	9.6	11.3	9.6
		PHV	10.6	9.3	11.0	9.6
	IDO	ANDV	8.6	8.3	10.0	10.3
		HTNV	9.0	7.6	8.0	9.3
		PHV	9.6	7.3	9.3	9.6
	CXCL10	ANDV	12.3	12.6	13.0	13.3
		HTNV	12.3	12.6	13.6	13.0
		PHV	12.3	12.0	13.3	12.6
	CXCL11	ANDV	13.6	13.3	13.6	13.6
		HTNV	13.3	12.6	13.0	12.6
		PHV	13.3	11.6	12.6	12.3
60	CCL5	ANDV	9.3	10.0	10.3	10.3
		HTNV	9.0	9.6	10.3	10.3
		PHV	8.3	9.3	10.3	9.6
	IFN-β	ANDV	7.3	7.3	10.3	8.6
		HTNV	7.6	7.0	9.0	8.6
		PHV	7.6	7.3	8.6	7.6
	IDO	ANDV	8.6	7.3	10.3	9.6
		HTNV	8.6	6.6	7.6	9.3
		PHV	9.0	7.3	9.3	9.6
	CXCL10	ANDV	12.0	12.3	13.3	13.0
		HTNV	11.6	12.0	13.3	13.0
		PHV	11.3	11.6	13.0	13.0
	CXCL11	ANDV	12.6	12.3	13.0	12.6
		HTNV	12.3	11.3	12.6	12.3
		PHV	11.6	11.6	12.0	12.0

Table S4. Analysis of variance of protein levels of CCL5, CXCL10, CXCL11, IDO, and IFN-β secreted by each of the four HLMVEC donors following infection by ANDV, HTNV, or PHV.

Protein	Variables	Df	Sum Sq	Mean Sq	F value
CCL5	Donor	3	21.3565279	7.1188426	11.4774868
	Virus	3	274.8916451	91.6305484	147.7330610
	Time point	1	21.3121337	21.3121337	34.3608852
	Replicate	2	1.7215510	0.8607755	1.3878014
	Residuals	84	52.1004981	0.6202440	NA
CXCL10	Donor	3	11.9307019	3.9769006	23.4688767
	Virus	3	565.8870005	188.6290002	1113.1559884
	Time point	1	0.3694529	0.3694529	2.1802516
	Replicate	2	0.0043903	0.0021952	0.0129544
	Residuals	85	14.4036103	0.1694542	NA
CXCL11	Donor	3	3.0108438	1.0036146	4.2374724
	Virus	3	524.8150407	174.9383469	738.6265808
	Time point	1	10.0610987	10.0610987	42.4800796
	Replicate	2	0.4437120	0.2218560	0.9367229
	Residuals	85	20.1316333	0.2368427	NA
IDO	Donor	3	17.7557884	5.9185961	31.8285961
	Virus	3	95.4190841	31.8063614	171.0459382
	Time point	1	1.1035385	1.1035385	5.9345293
	Replicate	2	0.0530458	0.0265229	0.1426329
	Residuals	85	15.8059335	0.1859522	NA
IFNB	Donor	3	14.0587286	4.6862429	32.6835094
	Virus	3	85.4521739	28.4840580	198.6578593
	Time point	1	56.6098194	56.6098194	394.8168319
	Replicate	2	0.5240646	0.2620323	1.8275054
	Residuals	82	11.7573640	0.1433825	NA

Table S5. Least mean estimate of log transformed protein levels of CCL5, CXCL10, CXCL11, IDO, and IFN-β secreted by each of the four HLMVEC donors following infection by ANDV, HTNV, or PHV.

Protein	Variable	Level	lsmean	SE	df	lower.CL	upper.CL
CCL5	Donor	2551	6.422330	0.1607591	84	6.013129	6.831531
		2559	5.184890	0.1644597	84	4.766270	5.603510
		2572	6.137328	0.1607591	84	5.728127	6.546529
		438Z013.1	6.178971	0.1644597	84	5.760351	6.597591
	Virus	ANDV	7.259582	0.1607591	84	6.850382	7.668783
		HTNV	7.066870	0.1683284	84	6.638402	7.495337
		PHV	6.506689	0.1607591	84	6.097489	6.915890
		Mock	3.090378	0.1607591	84	2.681177	3.499578
CXCL10	Donor	2551	7.646238	0.0840273	85	7.432407	7.860068
		2559	6.835044	0.0859593	85	6.616297	7.053791
		2572	7.131766	0.0840273	85	6.917936	7.345597
		438Z013.1	7.586329	0.0840273	85	7.372499	7.800160
	Virus	ANDV	8.794326	0.0840273	85	8.580496	9.008157
		HTNV	8.808009	0.0859593	85	8.589262	9.026756
		PHV	8.505416	0.0840273	85	8.291586	8.719247
		Mock	3.091626	0.0840273	85	2.877795	3.305456
CXCL11	Donor	2551	7.516668	0.0993401	85	7.263870	7.769466
		2559	7.434438	0.1016264	85	7.175821	7.693054
		2572	7.036365	0.0993401	85	6.783567	7.289163
		438Z013.1	7.307701	0.0994277	85	7.054680	7.560722
	Virus	ANDV	9.018513	0.0993401	85	8.765715	9.271311
		HTNV	8.640144	0.1016264	85	8.381528	8.898760
		PHV	8.321798	0.0993401	85	8.069000	8.574595
		Mock	3.314717	0.0994277	85	3.061696	3.567738
IDO	Donor	2551	5.683056	0.0880228	85	5.459058	5.907054
		2559	5.543995	0.0900465	85	5.314847	5.773143
		2572	4.798292	0.0880228	85	4.574294	5.022290
		438Z013.1	5.960967	0.0880228	85	5.736969	6.184965
	Virus	ANDV	6.315423	0.0880228	85	6.091424	6.539420
		HTNV	5.724507	0.0900465	85	5.495359	5.953655
		PHV	6.136171	0.0880228	85	5.912173	6.360169
		Mock	3.810211	0.0880228	85	3.586213	4.034209
IFNβ	Donor	2551	6.431325	0.0790894	82	6.229902	6.632749
		2559	5.649687	0.0790713	82	5.448309	5.851064
		2572	5.407521	0.0790894	82	5.206098	5.608945
		438Z013.1	5.755120	0.0790894	82	5.553697	5.956544
	Virus	ANDV	6.723792	0.0772934	82	6.526942	6.920642
		HTNV	6.273919	0.0790713	82	6.072541	6.475297
		PHV	6.186195	0.0772934	82	5.989345	6.383045
		Mock	4.059748	0.0833278	82	3.847530	4.271966

Table S6. Pairwise comparison of protein secretion levels induced by each virus following infection of each HLMVEC donor.

Protein	Variable	contrast	estimate	SE	df	t.ratio	p.value
CCL5	Donor	2551 - 2559	1.2374399	0.2299793	84	5.3806573	0.0000039
		2551 - 2572	0.2850019	0.2273478	84	1.2535947	0.5948066
		2551 - 438Z013.1	0.2433591	0.2299793	84	1.0581782	0.7157248
		2559 - 2572	-0.9524380	0.2299793	84	-4.1414070	0.0004680
		2559 - 438Z013.1	-0.9940808	0.2324005	84	-4.2774462	0.0002866
		2572 - 438Z013.1	-0.0416428	0.2299793	84	-0.1810721	0.9978758
	Virus	ANDV - HTNV	0.1927128	0.2327616	84	0.8279410	0.8410650
		ANDV - PHV	0.7528930	0.2273478	84	3.3116361	0.0073471
		ANDV - Mock	4.1692048	0.2273478	84	18.3384472	0.0000000
		HTNV - PHV	0.5601802	0.2327616	84	2.4066698	0.0835832
		HTNV - Mock	3.9764920	0.2327616	84	17.0839724	0.0000000
		PHV - Mock	3.4163118	0.2273478	84	15.0268111	0.0000000
CXCL10	Donor	2551 - 2559	0.8111938	0.1202064	85	6.7483394	0.0000000
		2551 - 2572	0.5144713	0.1188326	85	4.3293783	0.0002346
		2551 - 438Z013.1	0.0599084	0.1188326	85	0.5041409	0.9578866
		2559 - 2572	-0.2967225	0.1202064	85	-2.4684413	0.0723132
		2559 - 438Z013.1	-0.7512854	0.1202064	85	-6.2499603	0.0000001
		2572 - 438Z013.1	-0.4545629	0.1188326	85	-3.8252374	0.0013966
	Virus	ANDV - HTNV	-0.0136826	0.1202064	85	-0.1138257	0.9994683
		ANDV - PHV	0.2889101	0.1188326	85	2.4312365	0.0788864
		ANDV - Mock	5.7027006	0.1188326	85	47.9893616	0.0000000
		HTNV - PHV	0.3025927	0.1202064	85	2.5172757	0.0643842
		HTNV - Mock	5.7163831	0.1202064	85	47.5547194	0.0000000
		PHV - Mock	5.4137904	0.1188326	85	45.5581251	0.0000000
CXCL11	Donor	2551 - 2559	0.0822301	0.1421139	85	0.5786211	0.9382376
		2551 - 2572	0.4803029	0.1404881	85	3.4188162	0.0052569
		2551 - 438Z013.1	0.2089667	0.1405500	85	1.4867776	0.4499240
		2559 - 2572	0.3980727	0.1421139	85	2.8010817	0.0314296
		2559 - 438Z013.1	0.1267365	0.1422456	85	0.8909699	0.8095476
		2572 - 438Z013.1	-0.2713362	0.1405500	85	-1.9305309	0.2231544
	Virus	ANDV - HTNV	0.3783693	0.1421139	85	2.6624362	0.0450109
		ANDV - PHV	0.6967154	0.1404881	85	4.9592497	0.0000211
		ANDV - Mock	5.7037960	0.1405500	85	40.5819593	0.0000000
		HTNV - PHV	0.3183461	0.1421139	85	2.2400766	0.1208265
		HTNV - Mock	5.3254267	0.1422456	85	37.4382540	0.0000000
		PHV - Mock	5.0070806	0.1405500	85	35.6248964	0.0000000
IDO	Donor	2551 - 2559	0.1390608	0.1259221	85	1.1043399	0.6878590
		2551 - 2572	0.8847638	0.1244830	85	7.1075083	0.0000000
		2551 - 438Z013.1	-0.2779103	0.1244830	85	-2.2325168	0.1227918
		2559 - 2572	0.7457030	0.1259221	85	5.9219371	0.0000004
		2559 - 438Z013.1	-0.4169712	0.1259221	85	-3.3113414	0.0073253

		2572 - 438Z013.1	-1.1626742	0.1244830	85	-9.3400251	0.0000000
		ANDV - HTNV	0.5909158	0.1259221	85	4.6927078	0.0000597
		ANDV - PHV	0.1792515	0.1244830	85	1.4399681	0.4782614
		ANDV - Mock	2.5052117	0.1244830	85	20.1249333	0.0000000
	Virus	HTNV - PHV	-0.4116643	0.1259221	85	-3.2691970	0.0083243
		HTNV - Mock	1.9142959	0.1259221	85	15.2022187	0.0000000
		PHV - Mock	2.3259602	0.1244830	85	18.6849651	0.0000000
IFNB	Donor	2551 - 2559	0.7816387	0.1118701	82	6.9870201	0.0000000
		2551 - 2572	1.0238043	0.1116604	82	9.1689119	0.0000000
		2551 - 438Z013.1	0.6762051	0.1116604	82	6.0559087	0.0000002
		2559 - 2572	0.2421656	0.1118701	82	2.1647033	0.1418584
		2559 - 438Z013.1	-0.1054336	0.1118701	82	-0.9424642	0.7820806
		2572 - 438Z013.1	-0.3475992	0.1116604	82	-3.1130033	0.0133414
	Virus	ANDV - HTNV	0.4498727	0.1105737	82	4.0685327	0.0006165
		ANDV - PHV	0.5375969	0.1093094	82	4.9181227	0.0000260
		ANDV - Mock	2.6640437	0.1136565	82	23.4394332	0.0000000
		HTNV - PHV	0.0877242	0.1105737	82	0.7933551	0.8572676
		HTNV - Mock	2.2141710	0.1149711	82	19.2585085	0.0000000
		PHV - Mock	2.1264468	0.1136565	82	18.7094179	0.0000000

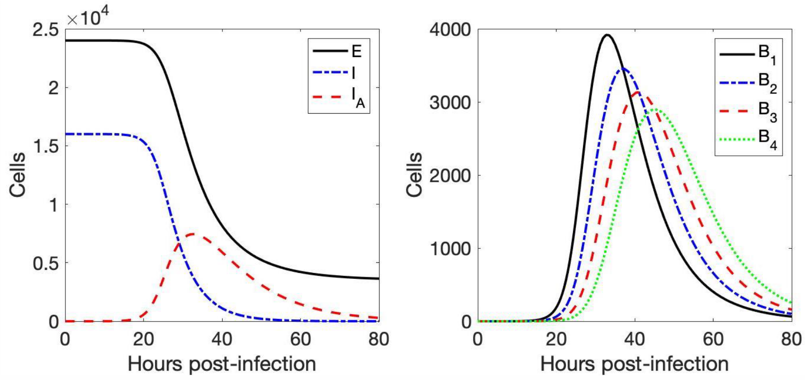

Figure S1. Graphs of the seven cell stages over time in the HLMVEC target-cell limited model (1).

Dynamics of model (1):

Based on the experimental results, the initial conditions are fixed at *E(0)*=24,000 cells and *I(0)*= 16,000 cells with other initial values in model (1) set to zero. It follows from the theory of differential equations that each of the seven variables in model (1) are nonnegative and bounded [1]. The uninfected endothelial cells *E* decrease over time as they transition to bystander cells $B_{1.}$ The activated infected cells I_A eventually stop signaling over time $I_A \rightarrow 0$ because the transition rate $f(t) \rightarrow 0$ and signaling decreases over time. Also, bystander cells B_1 through B_4 initially increase, then decrease to zero over time. The solution for each of the cell stages in the target-cell limited model (1) are graphed in the Figure S1

Reference

1. Brauer, F. and J.A. Nohel, *The Qualitative Theory Of Ordinary Differential Equations; An Introduction*. University mathematics series. 1969, New York,: W. A. Benjamin. xi, 314 p.

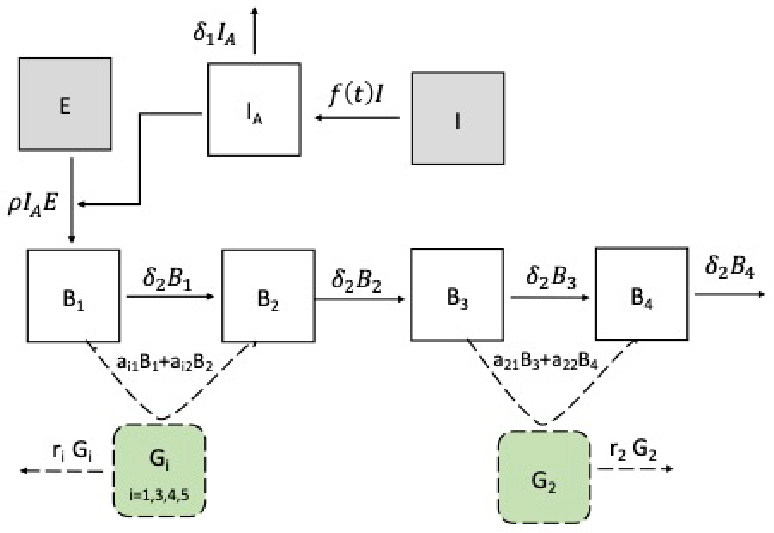

Figure S2. Compartmental diagram of models (1) and (3).

Compartmental diagram and discussion of models (1) and (3):

The dynamics of models (1) and (3) are illustrated in the compartmental diagram in Figure S2. The transition rates between the cell stages (compartments) in model (1) are denoted by arrows which show the direction of the rate of change between uninfected endothelial cells and bystander cells, E, B_1, B_2, B_3, B_4, and between infected and actively infected endothelial cells, I and I_A. Models (1) and (3) begin at time 0 with a portion of the 40,000 cells infected, $I(0)$=16,000, and the remaining cells uninfected, $E(0)$=24,000 (gray compartments). After a delay of approximately 24 hours, the infected endothelial cells are activated I_A at rate $f(t)I$ and begin signaling neighboring uninfected endothelial cells E (rate $\rho I_A E$) to bystander activation when gene expression levels of the chemokines and cytokines are upregulated, G_1 through G_5 (green compartments). The dashed curves indicate the rate of fold change of the intracellular gene expression levels within the bystander cells ($a_{1i}B_1+a_{2i}B_2$, i=1,3,4,5; $a_{12}B_3+a_{22}B_4$, i=2) and the rate of decrease in fold change ($r_i G_i$).To account for the time that gene expression levels reach their peak values within the bystander cells, the bystander cells are divided into four stages, B_1 to B_4, where B_1 represents the time of the earliest activation and B_4 the latest. Each stage has an average duration of four hours. The models follow the dynamics of the HLMVECs and gene expression levels from time 0 to 72 hours post-infection.

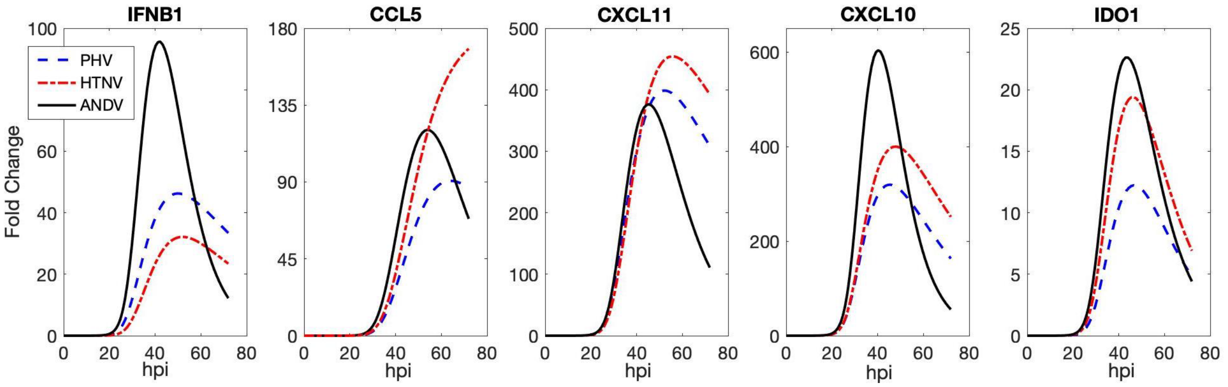

Figure S3. Comparison of PHV, HTNV, and ANDV infection on specific cytokine or chemokine upregulation. Fold change curves were fit to model (3) for IFNB1, CCL5, CXCL11, CXCL10 and IDO1 for the three viruses, PHV, HTNV, and ANDV. Fitted curves show continuous fold change over time.
